# Acute Inactivation of PSD-95 Destabilizes AMPA Receptors at Hippocampal Synapses

**DOI:** 10.1371/journal.pone.0053965

**Published:** 2013-01-16

**Authors:** Guillermo A. Yudowski, Olav Olsen, Hillel Adesnik, Kurt W. Marek, David S. Bredt

**Affiliations:** 1 Department of Anatomy & Neurobiology, School of Medicine, University of Puerto Rico, San Juan, Puerto Rico; 2 Institute of Neurobiology, University of Puerto Rico, San Juan, Puerto Rico; 3 Laboratory of Brain Development and Repair, The Rockefeller University, New York, New York, United States of America; 4 Department of Molecular & Cell Biology, University of California, Berkeley, California, United States of America; 5 NHLBI at National Institutes of Health, Bethesda, Maryland, United States of America; 6 Neuroscience at Johnson & Johnson, San Diego, California, United States of America; CNRS - Université Aix Marseille, France

## Abstract

Postsynatptic density protein (PSD-95) is a 95 kDa scaffolding protein that assembles signaling complexes at synapses. Over-expression of PSD-95 in primary hippocampal neurons selectively increases synaptic localization of AMPA receptors; however, mice lacking PSD-95 display grossly normal glutamatergic transmission in hippocampus. To further study the scaffolding role of PSD-95 at excitatory synapses, we generated a recombinant PSD-95-4c containing a tetracysteine motif, which specifically binds a fluorescein derivative and allows for acute and permanent inactivation of PSD-95. Interestingly, acute inactivation of PSD-95 in rat hippocampal cultures rapidly reduced surface AMPA receptor immunostaining, but did not affected NMDA or transferrin receptor localization. Acute photoinactivation of PSD-95 in dissociated neurons causes ∼80% decrease in GluR2 surface staining observed by live-cell microscopy within 15 minutes of PSD-95-4c ablation. These results confirm that PSD-95 stabilizes AMPA receptors at postsynaptic sites and provides insight into the dynamic interplay between PSD-95 and AMPA receptors in live neurons.

## Introduction

Chemical synapses are highly specialized sites for communication between neurons. On the presynaptic side, vesicles filled with neurotransmitter aggregate at the nerve terminal active zone. On the postsynaptic side, neurotransmitter receptors and signaling molecules align with sites of presynaptic vesicle release in an electron-dense specialization, the postsynaptic density (PSD). Multivalent scaffolding proteins assemble the neurotransmitter receptors and signaling molecules into organized signal transduction pathways.

One major family of postsynaptic scaffolding molecules is the membrane associated guanylate kinase proteins (MAGUKs). MAGUKs share a common core domain organization, consisting of three N-terminal PSD-95/Discs large/zona occludens-1 (PDZ) domains, a Src homology (SH3) domain, and a guanylate kinase (GK) domain [1]. PDZ domains often bind to the C-terminal tails of receptors, channels, and adhesion molecules [2]–[4]. In addition, PDZ domains may form dimers, as in the case of nNOS and the 2^nd^ PDZ domain of PSD-95 [5]. The SH3 and GK domains also bind receptors as well as other cytoplasmic molecules such as GKAP, MAP1A and SPAR1 [6].

The prototypical MAGUK, PSD-95, was originally identified as a core biochemical component of the PSD. Since its isolation, PSD-95 has been implicated in the trafficking and stabilization of numerous postsynaptic molecules including all three classes glutamate receptors (kainate, N-methyl-D-aspartate (NMDA), and alpha-amino-3-hydroxy-5-methyl-4-isoxazolepropionic acid (AMPA) type glutamate receptors. However, recent studies have demonstrated that PSD-95 may specifically mediate synaptic localization of only AMPA receptors [7]. Over-expression of PSD-95 in dissociated hippocampal neurons leads to a selective enhancement of AMPA receptors at the PSD [8] and in hippocampal slice cultures PSD-95 enhances AMPA receptor mediated synaptic responses without changing in NMDA responses [9], [10].

PSD-95 mediates these effects by binding to transmembrane AMPA receptor regulatory proteins (TARPs) [11]. TARPs and AMPA receptors directly interact through their transmembrane and other regions, while the binding between TARPs and PSD-95 occurs via the TARPs conserved C-termini and the first and/or second PDZ domain of PSD-95 [12]. Over-expression of TARPs lacking the PDZ ligand causes a specific loss of synaptic AMPA receptor responses, suggesting the TARP/PSD-95 interaction is critical for retention of AMPA receptors at the synapse [9], [13]. Stargazin (gamma-2) interaction with PDS-95 plays a key role in trapping AMPA receptors at synapses [14]. Recently, acute disruption of endogenous AMPAR-MAGUK interaction in cultured neurons was achieved by biomimetic divalent ligands, suggesting multivalent PDZ interactions [15]. However, several questions remain regarding the function of PSD-95 in AMPA receptor stabilization. For example, why do mice lacking PSD-95 display no gross changes in synaptic AMPAR expression? This might be explained by molecular compensation by other MAGUK isoforms, specifically PSD-93 and/or SAP-102. Furthermore, NMDA receptors robustly bind to PSD-95 but are unaffected by its over-expression [8]. Does this mean that NMDA receptors are anchored by other scaffold proteins at postsynaptic sites or are they not as dynamically regulated as AMPAR receptors? Is-PSD-95 required or essential for AMPA receptor stabilization at synapses?

To address these questions, we acutely disrupted PSD-95 in live cultures utilizing flourescein assisted light inactivation (FALI) and measured endogenous AMPA receptor localization [16]–[18]. A tetracystein motif was fused in frame into PSD-95 (PSD-95-4c), and this enables binding of: 4′,5′-bis(1,3,2-dithioarsolan-2-yl) fluorescein (FlAsH). Additionally, our PSD-95-4c contained a monomeric red fluorescence protein domain to track real-time expression. Following FlAsH binding, illumination photoinactivates the target with a damage radius of 40 Å [19], [20]. Endogenous GluR2 subunits of AMPA receptors disappear within 15 minutes of photoinactivation of PSD-95-4c in hippocampal neurons. In contrast, we did not see changes in surface staining of the NMDA receptor subunit NR1. Our experiments demonstrate that PSD-95 is necessary for endogenous AMPA receptor stability at the plasma membrane in hippocampal pyramidal cells. These data extend the role for PSD-95 as a necessary scaffolding protein at postsynaptic sites to maintain AMPA receptors at synapses. They also indicate that NMDA receptors are stabilized by a different scaffold protein.

## Materials and Methods

### PSD-95 Constructs and Viral Vectors

PSD-95-4c was generated with the FlAsH epitope (GGGACCPGCCGGGA) between the first two PDZ domains. This construct also carried a C-terminal RFP tag. The internal tetracysteine epitope was inserted by performing PCR in both directions from the site of the tag and then cloning the fragments together. The first fragment was created using a primer to the 5′ end of the gene with an engineered HindIII restriction site (‘HindIII primer’: 5′-CGT CGT AAG CTT CCC AAC ATG GAC TGT CTC TGT ATA GTG- 3′) and a primer containing half of the tetracysteine tag, a NarI restriction site and sequence specific to the tag location (5′-TAG TCT GGC GCC GCC GCC TGG GGG TTT CCG GCG CAT G-3′). The second fragment used a 3′ primer with an EcoRI restriction site (‘EcoRI primer’: 5′-CCG GAA TTC GAG TCT CTC TCG GGC TGG-3′) and a primer containing the other half of the tetracysteine tag, a NarI restriction site and sequence specific to the tag location (5′-TGA CTC GGC GCC TGC TGC CCC GGC TGC TGC GGC GGC GGC GCT GCC GAA AAG GTC ATG GAG ATC-3′). The first fragment was digested with HindIII/NarI and the second fragment was digested with EcoRI/NarI. The fragment was then ligated with a HindIII/EcoRI fragment of GW1 [21]. A monomeric RFP tag was then cloned into the EcoRI site to create GW1-PSD-95-4c-RFP. Viral vectors were created by cloning into pSCA1 [22].

#### Cell cultures and transfection

Neuronal cultures were prepared from hippocampi of E18/E19 rats as described [23] In brief, hippocampi were dissociated by enzyme digestion with papain followed by brief mechanical trituration. Cells were plated on poly-D-lysine (Sigma, St. Louis, MO) treated glass coverslips (12 mm in diameter) and maintained in Neurobasal media (Life Technologies, Rockville, MD) supplemented with B27, penicillin, streptomycin, and L-glutamine as described [24]. COS7 cells were cultured as described [25] and transiently transfected with Lipofectamine 2000 (Invitrogen). All experiments were performed 48–72 hours after transfection. All animals were housed, cared for, and experiments conducted in accordance with approved protocols from the University of California San Francisco IACUC committee approval number: AN075386 and the University of Puerto Rico IACUC committee, approval number: A9200211.

#### Immunostainings and antibodies

Immunocytochemistry on low density cortical or hippocampal neuron cultures (5×10^4^/cm^2^) was performed at 21–25 DIV. Cover slips were removed from culture wells and fixed in 4% paraformaldehyde for 10–15 min. Cells were washed with phosphate-buffered saline containing 0.1% Triton-X-100 (PBST) for permeabilized cells or without Triton-X-100 for non-permeabilized cells. Coverslips were incubated for 1 h at RT with primary antibodies (GluR2 1∶500, TfR 1∶250) washed in PBST, and incubated for 1 h at room temperature with secondary antibodies (1∶2000 for Alexa 488 [Molecular Probes] or 1∶500 for Cy3-labeled anti-mouse [Jackson ImmunoResearch]). Coverslips were then washed with PBS and mounted on slides (Frost Plus; Fisher Scientific) with Fluoromount-G (Southern Biotechnology Associates), and images were taken under fluorescence microscopy with 100X objective on a Zeiss inverted microscope (Thornwood, NY). Monoclonal mouse anti-GluR2, cat# MAB397 was purchased from Millipore (Billerica, MA) monoclonal antibody anti-human transferrin receptor (cat# CD71-H300) from Santa Cruz Biotechnology (Santa Cruz,CA). Anti-NR1 from BD-bioscience, cat# 556308 (San Jose, CA). Polyclonal antibody anti Kv1.4 was a gift from L. Jan, UCSF.

#### FlAsH incubation and photoinactivation

Cells were incubated with 0.5–1 µM FlAsH-EDT_2_ (Invitrogen, Carlsbad, CA), and 15 µM EDT in 1× Hank’s Balanced Salt Solution (HBSS, Invitrogen Carlsbad, CA) supplemented with D^+^ glucose (1 g/l) for 5 to 10 minutes at 37°C. Preparations were then rinsed for 15 min twice in HBSS, 250 µM EDT to remove excess FlAsH. Photoinactivation was executed for 2 minutes using a 100 W mercury lamp. COS7 cells were then incubated for 15 minutes at 37°C, fixed and stained. For live confocal analysis, cells were loaded with FlAsH and washed before transferring them into a heated chamber for photoinactivation and imaging.

### Fluorescent Analysis and Live Confocal Imaging

Confocal images used laser scanning fluorescent microscope LSM510 (Zeiss, Thornwood, NY) equipped with 63X and 100X objectives. Images are presented as single sections. Colocalization was determined in automatically thresholded images for light objects by calculating the average pixel intensity of puncta for PSD-95 and comparing it with the indicated receptor pixel intensity in the same field. Measurements were done only in dendrites using the image system analysis software MetaMorph (Molecular Devices, Sunnyvale, CA, USA). Results represent means ± SEM of fluorescence intensity. Statistical tests were calculated according to standard algorithms using GraphPad Prism software (San Diego, CA) with a significance threshold of p<0.05. Matched samples were analyzed utilizing the Wilcoxon T test.All imaging was done using a stage and objective heated to 37°C (Bioptech, San Diego, CA).

### Electrophisiology

Recordings were performed at room temperature with an Axopatch-1B amplifier and patch pipettes of 35 MΩ. Series resistances ranged between 10 and 20 MΩ. The external solutions contained 140 mM NaCl, 2.4 mM KCl, 10 mM HEPES, 10 mM glucose, 4 mM CaCl2, 4 mM MgCl2, 0.1 mM picrotoxin, and 0.005 mM TTX (pH 7.27). The internal solution contained 107.5 mM d-glucoronicacid, 20 mM HEPES, 0.2 mM EGTA, 8 mM NaCl, 10 mM TEA-Cl, 4 mM Mg-ATP, 0.3 mM Mg-GTP, 5 mM QX-314, and 0.1 mM spermine (pH 7.2).

mEPSCs (about 100 events per cell) were automatically detected using in-house software. Picrotoxin and TTX were from Sigma. Neurons were used 16–24 hours after infection. Statistical analysis used the Kolmogorov-Smirnov test.

## Results

### Fali Disrupts Kv 1.4/PSD-95 Clustering in COS Cells

**Figure 1:**
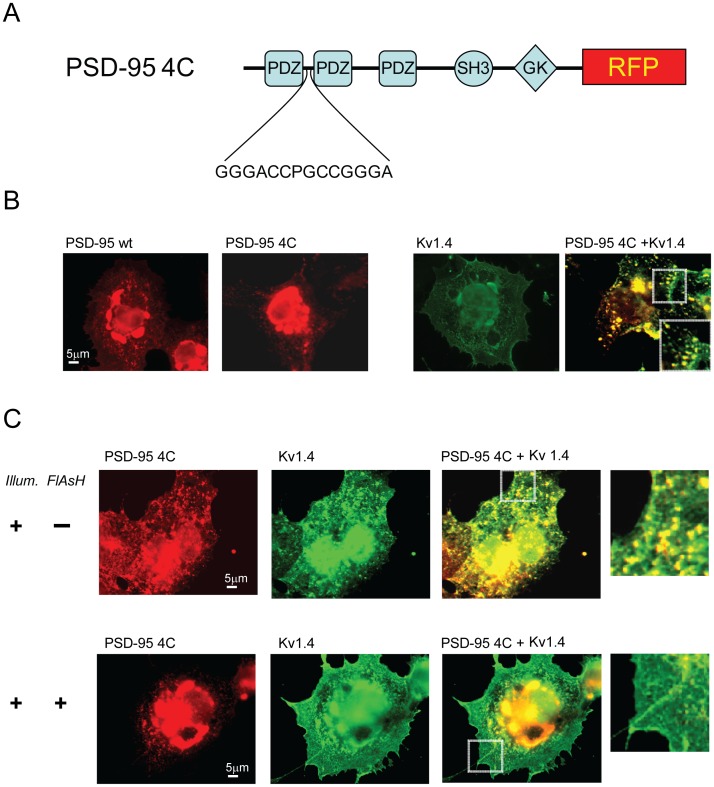
Fluorescein-assisted light inactivation (FALI) of PSD-95-4c redistributes Kv1.4 in COS7 cells. (A) A tetracysteine motif was engineered between PDZ1 and PDZ2 of PSD-95 for binding of FlAsH. (B) COS7 cells were transfected with PSD-95 or PSD-95-4c or co-transfected with PSD-95-4c and Kv1.4. Immunofluorescence experiments show perinuclear distribution of labeled proteins in single transfected cells. Double transfected cells show clustering of labeled proteins (see insert). (C) COS7 cells were co-transfected with PSD-95-4c and Kv1.4 for three days. Cells were then incubated in the presence or absence of 1 µM FlAsH-EDT_2_ at 37°C, washed and illuminated for 2 minutes. After illumination, cells were incubated at 37° for 15 minutes before fixation and immunostaining.

The FALI methodology allows specific ablation of proteins containing the FlAsH binding motif. A tetracysteine motif that binds the membrane-permeable fluorescein derivative FlAsH was engineered between the first and second PDZ domains of a PSD-95 fusion to RFP (PSD-95-4c, Fig. 1A). To assess the functionality of PSD-95-4c, we tested its ability to form clusters when co-expressed with Kv 1.4. COS7 cells were single transfected with PSD-95 (wt), PSD-95-4c or Kv1.4, or co-transfected with both PSD-95-4c and Kv1.4. Three days after transfection, cells were fixed, permeabilized and visualized by immunofluorescence.

Expression of Kv1.4, PSD95 or PSD-95-4c alone resulted in a perinuclear localization pattern for each protein (Fig. 1B). In contrast, co-transfection of PSD-95-4c and Kv1.4 caused the proteins to cluster throughout the cytoplasm (Fig. 1B). This pattern was similar to that seen with wild type PSD-95 and Kv1.4 co-expression [26].

**Figure 2:**
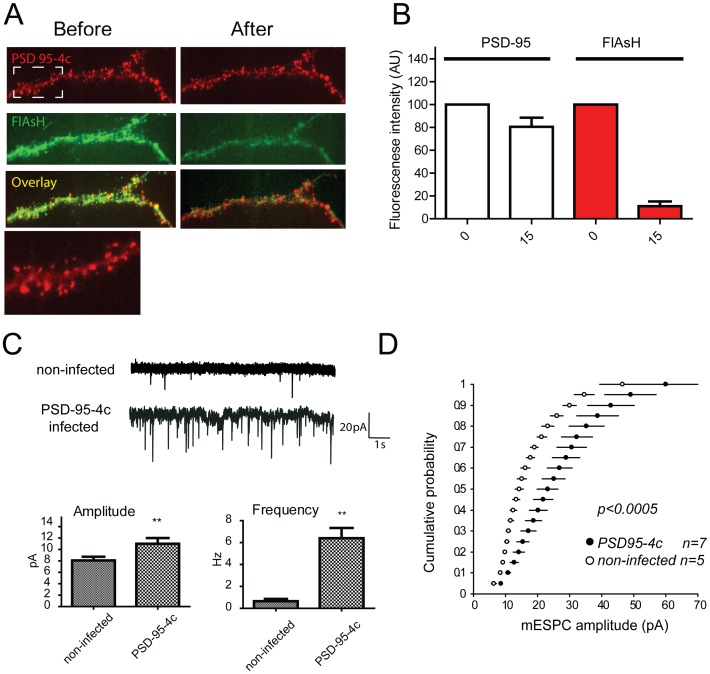
PSD-95-4c localizes at synaptic spines and increases EPSCs. Dissociated hippocampal neurons DIV 21∼25 were infected with a Semliki forest virus for over-expression of PSD-95-4c. (A) PSD-95-4c localized to synaptic spines much like wt-PSD-95. Incubation with FlAsH shows localization of the fluorescein derivative at PSD-95-4c enriched spines. (B) PSD-95-4c and FlAsH fluorescence were quantified before and after FALI. (C) Whole-cell recordings were measured 16–24 hours after infection. PSD-95-4c significantly increased the amplitude and frequency of AMPA receptor-mediated mEPSCs compared to control indicating functional PSD-95-4c construct by the recruitment of additional AMPA receptors to synapses. (D) Cumulative probability distribution of mEPSC amplitude from control and PSD-95-4c infected neurons.

Next, we determined whether FALI disrupts PSD-95-4c/Kv1.4 clustering. COS cells were loaded with FlAsH, washed and illuminated for photoinactivation and subsequently incubated for sixty minutes at 37°C before fixation and immunostaining. Illumination protocol dramatically reduced FlAsH fluorescence signal in COS cells by 93%, similar to the reduction observed in neurons (Fig. 2B). This allowed us to investigate Kv1.4 by immunohistochemistry in the green channel without a significant signal from the FlAsH dye. Omitting FlAsH treatment from illuminated cells served as a control for non-specific effects of our illumination protocol (Fig. 1C, top).

In contrast, illumination of FlAsH loaded cells expressing PSD-95-4c and Kv 1.4 redistributed PSD-95-4c/Kv 1.4 localization from clusters to a perinuclear distribution resembling singly transfected cells (Fig. 1C, bottom and inserts).

Live cell analysis in COS cells co-expressing RFP-PSD-95-wt and Kv 1.4-CFP did not show redistribution of clusters after FlAsH incubation and illumination, demonstrating the specificity of the redistribution effect to the tetracysteine-containing construct (Figure S1).

Taken together these results demonstrate that the PSD-95-4c forms clusters with Kv1.4 similar to those observed with the wild type PSD-95 and this localization is specifically ablated in the PSD-95 tetracysteine-containing motif after FlAsH incubation and illumination.

### PSD 95-4c Localizes at Spines and Increases EPSCs in Infected Hippocampal Neurons

In dissociated neurons, transfected PSD-95 specifically localizes to dendritic spines, selectively increases the number of synaptic AMPARs and dramatically enhances AMPA mediated excitatory postsynaptic currents [9]. To ensure PSD-95 functions were retained by PSD-95-4c, we first investigated the cellular localization of over-expressed PSD-95-4c. Hippocampal cells were isolated and infected (DIV 21–25) with PSD-95-4c adenovirus. Similar to wild type, PSD-95-4c localized to dendritic spines (Fig. 2A, PSD-95 and insert). Infected neurons incubated with FlAsH showed increased colocalization of FlAsH with PSD95-4c protein (Fig. 2A). Illumination decreased FlAsH fluorescence ∼93%, whereas RFP intensity decreased ∼22% compared to control (Fig. 2A, B). This shows that FALI decreased FlAsH signal to almost undetectable levels whereas RFP intensity decreased modestly with no apparent changes in PSD95-4c distribution or spine morphology.

PSD-95-4c function was also assessed by measuring excitatory postsynaptic currents in dissociated hippocampal neurons infected with PSD-95-4c. Cells over-expressing PSD-95-4c showed enhanced spontaneous and evoked excitatory postsynaptic currents (Fig. 2 C, D). Over-expression of PSD-95-4c increased amplitude and frequency as previously observed with PSD-95 GFP in pyramidal neurons [8].Together, these results indicate that PSD-95-4c localizes to dendritic spines and enhances AMPA receptor mediated excitatory postsynaptic currents to the same extent as wild type PSD-95-GFP.

### Photoinactivation of PSD-95-4c in Hippocampal Neurons Decreases GluR2 but not NR1 or TfR Surface Staining

**Figure 3:**
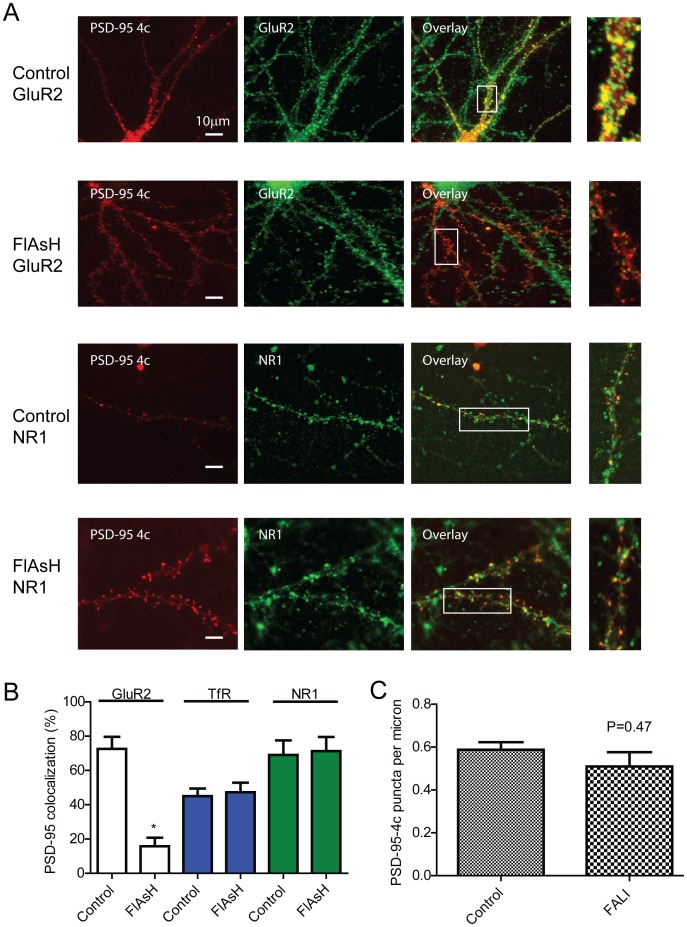
Photoinactivation of PSD-95-4c decreases GluR2 staining with no changes in NR1 or TfR staining. (A) Hippocampal neurons were infected DIV 21–25 with PSD-95-4c. 48 to 72 hours after infection, neurons were incubated with FlAsH-EDT_2_ for 5 minutes (FlAsH) washed and photo-inactivated. Controls were illuminated following the same protocol but without FlAsH-EDT_2_ incubation (control). After illumination/photoinactivation, neurons were incubated 15 minutes at 37°C, fixed and immunostained for GluR2 and NR1 in non-permeablized conditions. (B) Colocalization of PSD-95-4c with surface receptors. Only GluR2 in the FlAsH group showed a significant decrease (16% ±5 *n* = 7). (C) PSD-95-4c distribution was not changes after FALI. Clusters of immunofluorescence of PSD-95-4c were measured before and after FALI protocols. Quantification of PSD-95-4c puncta per micron did not show a significant difference between groups (n = 7 neurons, 262 and 211 clusters for control and FALI respectively. P = 0.47).

To study the acute effects of PSD-95 disruption, hippocampal neurons were infected (DIV 21–25) with PSD-95-4c. 48 to 72 hours after infection, cells were incubated with FlAsH, illuminated, incubated for 15 minutes and then fixed under non-permeabilizing conditions. In the absence of FlAsH, illuminated neurons show PSD95-4c co-localized with GluR2 in dendritic spines (76%±5, *n* = 6, Fig. 3A and B). However, cells incubated with FlAsH and illuminated, show a significant decrease in PSD-95-4c and GluR2 colocalization (16%±5 *n* = 7, Fig. 3A top and B). Uninfected neighboring dendrites show punctate GluR2 staining, indicating a specific effect of photoinactivation. We found no difference in NR1 staining between infected and non-infected cells after illumination in the presence or absence of FlAsH 44%±4.5 vs 49%±5.6 (Fig. 3A bottom and B). Transferrin receptor surface staining intensity also did not change in photoinactivated samples (Fig. 3B), indicating that FALI specifically inactivated PSD-95-4c without affecting global membrane integrity. To further evaluate the effect of FALI, we quantified the number of PSD-95 puncta per micron before and after the ablation protocol (Fig. 3C). This analysis indicated that there is no significant difference in the number or location of PSD-95 molecules between treatments and suggests that the decrease observed in GluR2 staining is specifically due to the effect of photoinactivation on receptor stability at the cell surface.

**Figure 4:**
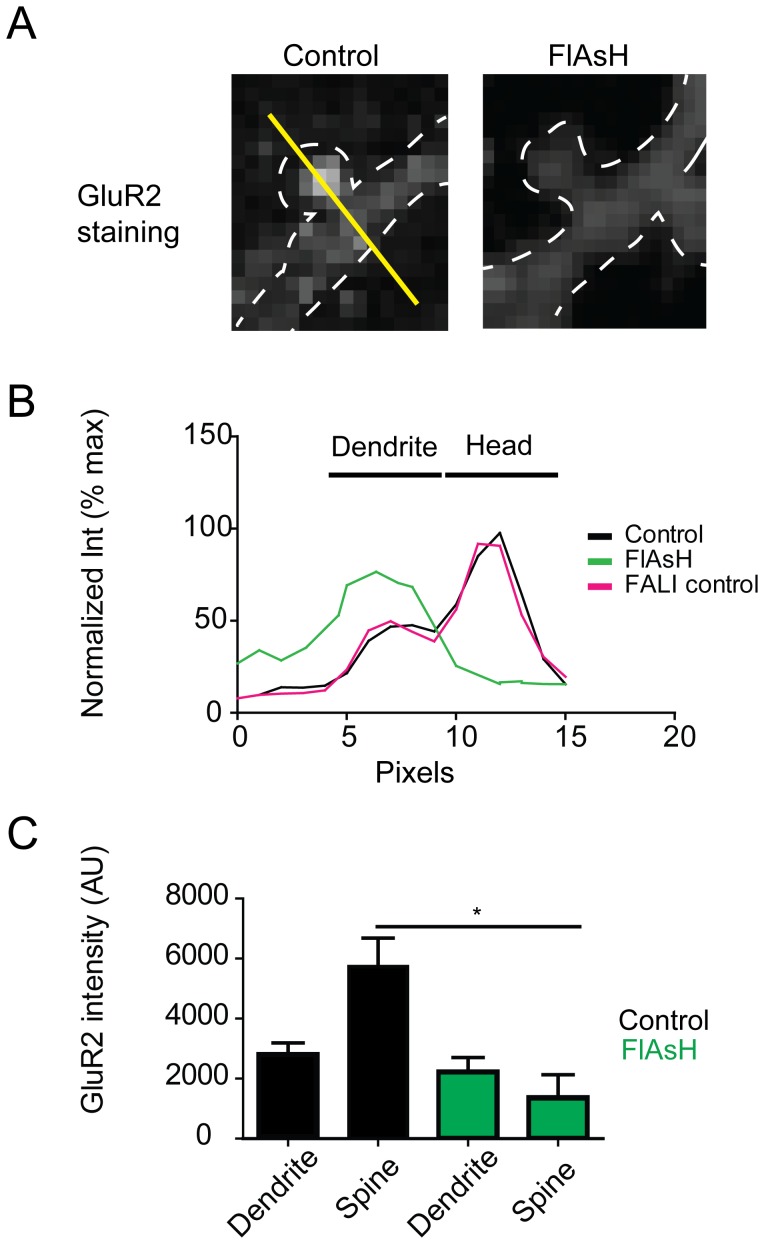
FALI selectively decreases GluR2 immunostaining in spines. (A) Hippocampal cells were infected with PSD-95-4c and FALI experiments were performed as described. GluR2 localization was measured by immunostaining in control and FlAsH treated cells. Line-scans were drawn through spines and dendrites (yellow line) to measure fluorescent intensity in controls before and after FALI. (B) Fluorescence intensities were normalized to the maximum intensity and plotted versus distance in control (no illumination), FALI control (illumination, no FlAsH) and FlAsH cells. (C) GluR2 intensity was significantly reduced in spines after FALI when compared to spines in control experiments (n = 4 cells in all conditions, 43 spines for control and 35 for FALI).

Next, to investigate the decreased in GluR2 immunostaining after FALI in more detail, we analyzed spine and dendrite intensities before and after PSD-95-4c ablation. Line-scans drawn through spines into dendrites (Fig. 4A) showed a significant decrease in GluR2 immunofluorescence intensity only after FALI (Fig. 4B). Illumination by itself did not significantly change the GluR2 fluorescent intensity measured in spines and dendrites when compared to control staining. Further analysis showed a statistical significant decreased in GluR2 fluorescence at spines when compared to controls or illuminated samples. GluR2 fluorescence at the dendrite did not show a significant decrease when compared to controls, suggesting a specific effect of FALI on GluR2 at spines.

### Live Imaging of GluR2

**Figure 5:**
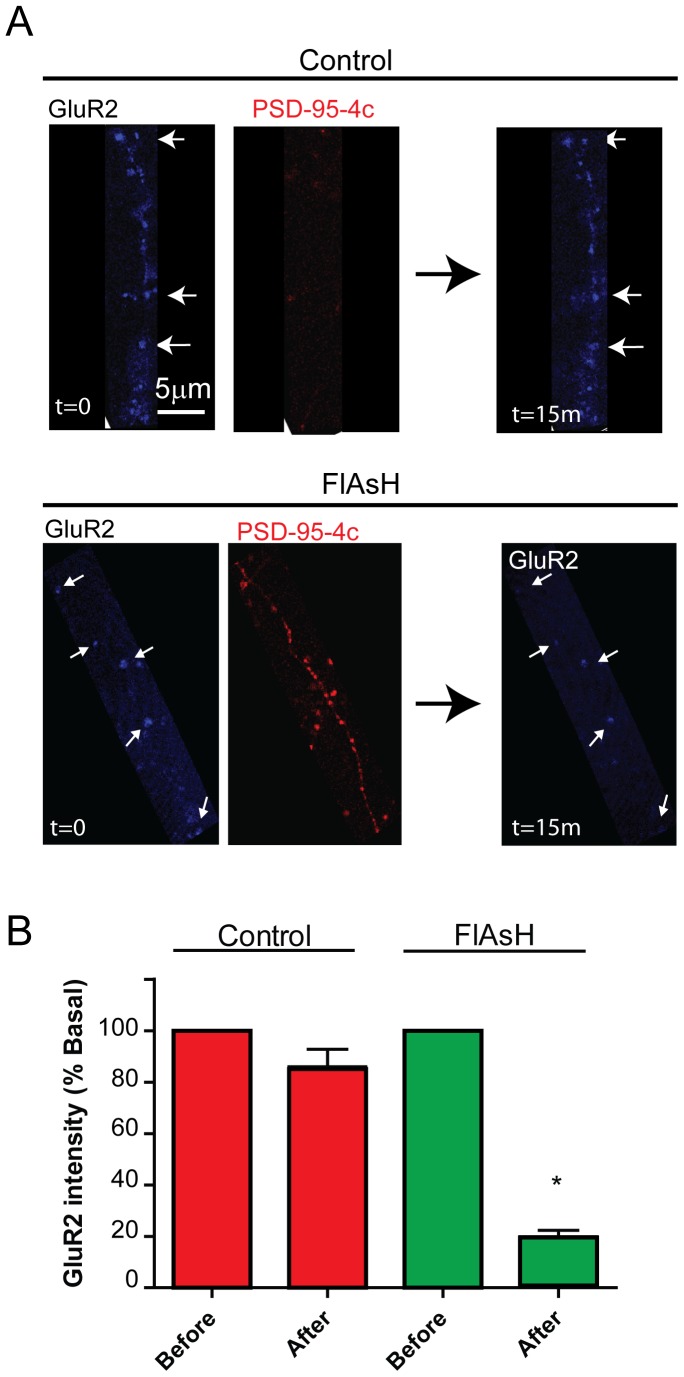
FALI induced rapid reduction of synaptic GluR2 staining. (A) Hippocampal cells were infected with PSD-95-4c and stained with Alexa647 conjugated anti GluR2 monoclonal antibody for confocal live cell imaging. Cells were incubated with FlAsH-EDT2, washed and mounted in a closed chamber at 37°C. Neighboring infected and non-infected dendrites were imaged for direct comparison. Images were taken before (t = 0 min) and 15 minutes after FALI protocol (t = 15 min). GluR2 are indicated by arrows. (B) Quantification of GluR2 surface staining intensities before and after FALI. Non-infected dendrites showed no significant decrease in GluR2 signal intensity (86%±7, *n* = 42). By contrast, infected dendrites showed a marked decreased compared to the initial intensity (20±2%, *n* = 35).

We monitored GluR2 expression using live imaging of hippocampal neurons (DIV21). 48–72 hours after infection, cells were incubated with FlAsH, washed and transferred to a heated chamber. Cells were imaged before (t = 0) and fifteen minutes after photoinactivation (t = 15). Figure 5 A shows a representative experiment of neighboring infected and non-infected dendrites with PSD-95-4c in red, GluR2 labeling in blue and FlAsH staining in green.

Non-infected (top) and infected (bottom) neighboring dendrites are shown before (t = 0 min) and after photoinactivation (t = 15 min).

After photoinactivation, total GluR2 surface staining from infected dendrites decreased to 20% ±2 compared to initial intensities (infected before illumination). In contrast, GluR2 intensity from non-infected neighboring synapses decreased to 86% ±7. GluR2 intensity was measured in 5 separate experiments totaling 35 measurements for infected GluR2 puncta and 42 for non-infected (Fig. 5 B). Discrete PSD-95-4c labeling in the control panel originates from a transecting dendrite.

These data demonstrate that acute photoinactivation of PSD-95-4c reduces GluR2 subunit surface staining within 15 minutes.

## Discussion

AMPA receptors are highly dynamic whereas NMDA receptors are stable at synaptic membranes [4], [27]. PSD-95 binds both receptors but over-expression of PSD-95 induces maturation of excitatory synapses by enhancing AMPA receptor-mediated synaptic responses with no change in NMDA receptor responses [8]. In this study, FALI was utilized to acutely probe the stabilizing effect of PSD-95 on AMPA and NMDA receptors.

Over-expressed PSD-95-4c showed similar synaptic distribution when compared to wild type PSD-95.Increased AMPA receptor-mediated mEPSCs frequency andamplitude was also observed. These data demonstrate that PSD-95-4c targets correctly and retains normal functional. When infected cells were illuminated for photoinactivation, FlAsH staining intensity decreased to almost undetectable levels. The intensity of RFP on the same PSD-95-4c construct decreased only modestly (to ∼80% of control) indicating a specific effect of the illumination on the fluorescein derivative.

AMPA receptors closely co-localized with PSD-95-4c as described before for PSD-95 wt. When cells infected with PSD-95-4c were photoinactivated and incubated at 37°C for 15 minutes and were fixed for surface staining, GluR2 intensity decreased to ∼10% of control, whereas NR1 or transferrin receptor staining was unaffected. Localization or number of PSD-95-4c molecules did not change between conditions supporting the specific effect of FALI on the surface localization of GluR2. Spine/dendrite intensity measurements indicate that FALI induced a significant decrease of GluR2 immunostaining at the spine when compared to the dendrite.

Live cell imaging allowed measuring GluR2 receptor intensities in identified dendrites before and after photoinactivation. These experiments also revealed a dramatic decrease in GluR2 staining after FALI (to ∼20% of the original level) further supporting the results obtained in fixed cells.

FALI is a powerful method to investigate acute loss of protein function and it has been extensively used in drosophila neuromuscular junction preparations but less so in dissociated neurons or cell cultures [18], [20]. In our experiments we had to perform extensive washes to significantly remove non-specific staining of the FlAsH dye. However, we did not see non-specific effects of this excess of FlAsH reagent in our controls. We believe that acute inactivation of proteins in vivo is a powerful approach to investigate their function, however further technical developments are needed to fully exploit the potential of FALI.

Our work indicates that acute disruption of PSD-95 by FALI, decreased endogenous synaptic GluR2 in cultured hippocampal neurons within 15 minutes of PSD-95 ablation. Reduction in receptor expression was specific to GluR2 without significant changes in NR1 receptors or Transferrin receptors. This effect underlies the dynamic role of PSD-95 as a synaptic scaffold for GluR2 containing receptors. Our data provides further evidence of the dominant role of PSD-95 in AMPA receptor synaptic stabilization in living neurons.

## Supporting Information

Figure S1 **Tetracysteine motif is required for FALI of PSD-95.** COS cells expressing RFP-PSD-95-wt and Kv 1.4-CFP show perinuclear clusters highly co-localized as observed in Figure 1. Cells were incubated with FlAsH and illuminated for 2 minutes following protocols as described above. Illumination protocol did not have an effect on the distribution of PSD-95/Kv1.4 clusters.
(TIF)
