# Supplementary figures and images for: Acute Inactivation of PSD-95 Destabilizes AMPA Receptors at Hippocampal Synapses
Source: PLoS One. 2013 Jan 16;8(1):e53965. doi: 10.1371/journal.pone.0053965 (PMC3546964; doi:10.1371/journal.pone.0053965)

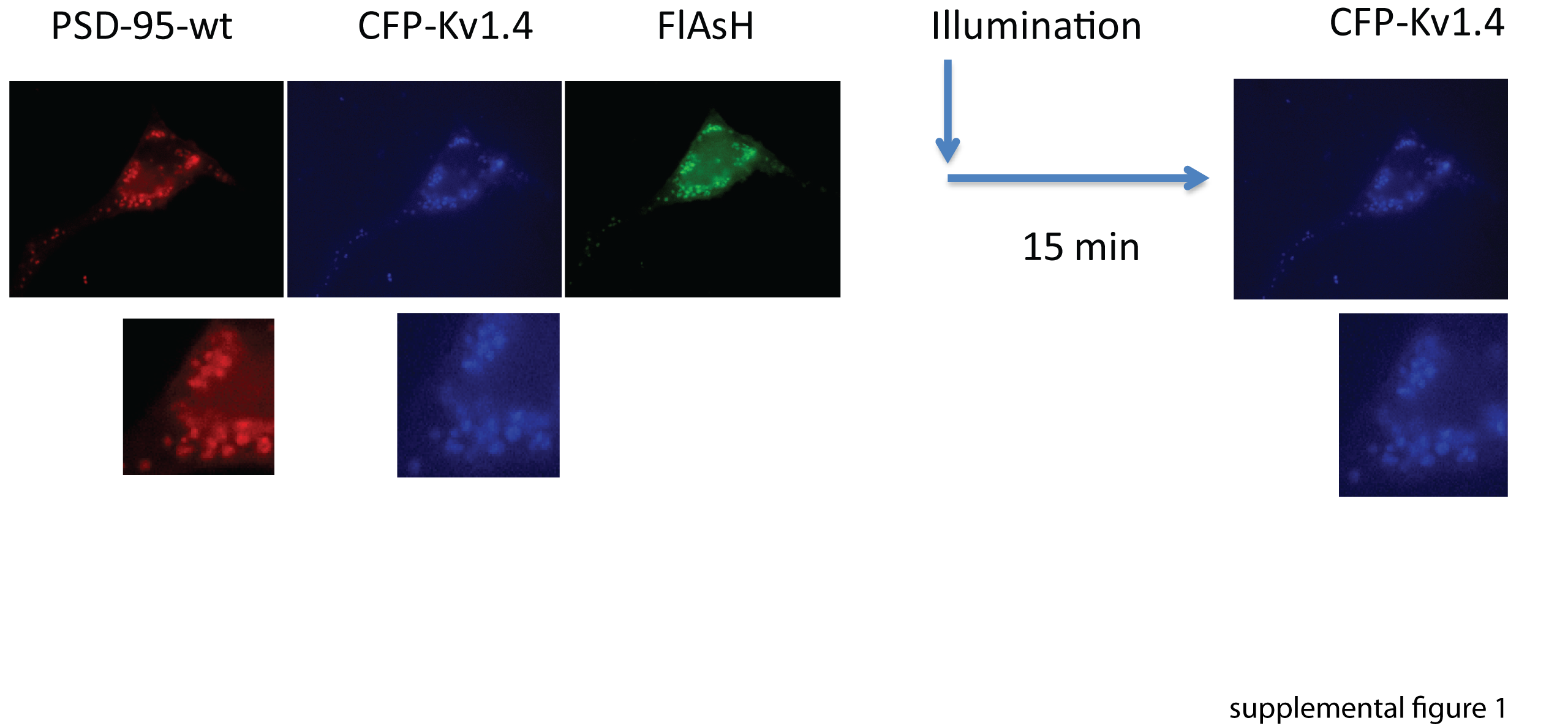

Supplement: Figure S1 — Tetracysteine motif is required for FALI of PSD-95. COS cells expressing RFP-PSD-95-wt and Kv 1.4-CFP show perinuclear clusters highly co-localized as observed in Figure 1. Cells were incubated with FlAsH and illuminated for 2 minutes following protocols as described above. Illumination protocol did not have an effect on the distribution of PSD-95/Kv1.4 clusters. (TIF) [file pone.0053965.s001.tif]
